# A long non-coding RNA with important roles in the carcinogenesis

**DOI:** 10.3389/fcell.2022.1037149

**Published:** 2022-11-16

**Authors:** Soudeh Ghafouri-Fard, Arian Askari, Bashdar Mahmud Hussen, Mohammad Taheri, Majid Mokhtari

**Affiliations:** ^1^ Department of Medical Genetics, Shahid Beheshti University of Medical Sciences, Tehran, Iran; ^2^ Phytochemistry Research Center, Shahid Beheshti University of Medical Sciences, Tehran, Iran; ^3^ Department of Pharmacognosy, College of Pharmacy, Hawler Medical University, Erbil, Iraq; ^4^ Center of Research and Strategic Studies, Lebanese French University, Erbil, Iraq; ^5^ Urology and Nephrology Research Center, Shahid Beheshti University of Medical Sciences, Tehran, Iran; ^6^ Institute of Human Genetics, Jena University Hospital, Jena, Germany; ^7^ Skull Base Research Center, Loghman Hakam Hospital, Shahid Beheshti University of Medical Sciences, Tehran, Iran

**Keywords:** lncRNA, TMPO-AS1, cancer, biomarker, expression

## Abstract

Long non-coding RNAs are demonstrated to contribute to carcinogenesis. TMPO Antisense RNA 1 (TMPO-AS1) is an example of lncRNAs with crucial roles in this process. This lncRNA serves as a sponge for miR-320a, miR-383-5p, miR-329-3p, miR-126, miR-329, miR‐199a‐5p, miR-577, miR-4731-5p, miR-140-5p, miR-1179, miR-143-3p, miR-326, miR-383-5p, let-7c-5p, let-7g-5p, miR-199a-5p, miR-200c, miR-204-3p, miR-126-5p, miR-383-5p, miR-498, miR-143-3p, miR-98-5p, miR-140 and miR-143. It can also affect activity of PI3K/Akt/mTOR pathway. The current review summarizes the role of TMPO-AS1 in the carcinogenesis and assessment of its potential as a marker for certain types of cancers.

## Introduction

Long non-coding RNAs (lncRNAs) are a class of RNAs with sizes more than 200 nt and some similar features with mRNAs, yet they do not encode large polypeptides. These transcripts have critical functions in the embryonic development (Kung et al., 2013), DNA damage response (Thapar, 2018; Ghafouri-Fard et al., 2021) and carcinogenic processes (Schmitt and Chang, 2017; Ghafouri-Fard et al., 2020). Based on the latest reports from GENCODE and FANTOM projects, there are approximately 18,000 and 28,000 lncRNA genes, respectively (Hon et al., 2017; Frankish et al., 2019). The role of these transcripts in the development of human disorders, particularly cancers is being elucidated in recent years. The vast majority of investigations are focused on identification of the impact of lncRNAs in the development of cancers, since cancers constitute a major cause of mortality. In fact, lncRNAs can affect all features of cancer development, including cell proliferation/differentiation, cell cycle transition, metastatic ability and invasiveness of cancer cells, epithelial-mesenchymal transition (EMT) and angiogenesis (Ghaforui‐Fard et al., 2019; Ghafouri-Fard and Taheri, 2019).

TMPO Antisense RNA 1 (TMPO-AS1) is an example of lncRNAs with crucial roles in the carcinogenesis. Genomic location for this lncRNA is chr12:98,510,417–98,516,454 (GRCh38/hg38), minus strand. Expression of this lncRNA has been appraised in numerous types of cancers, revealing its important roles in the oncogenesis. The current review aims at identification of the impact of TMPO-AS1 in the carcinogenesis and evaluation of its potential as a marker for certain types of cancers.

### Cell line studies

TMPO-AS1 has been noticeably upregulated in nasopharyngeal cancer cells. TMPO-AS1 silencing has restrained aggressive behaviors of these cells, while its over-expression has led to the opposite results. Mechansitically, TMPO-AS1 acts as a molecular sponge for miR-320a, leading to up-regulation of the mRNA target of miR-320a, i.e. SOX4. Taken together, TMPO-AS1/miR-320a/SOX4 axis has been shown to enhance progression of nasopharyngeal carcinoma (Xing et al., 2021).

This lncRNA has also been shown to be up-regulated in bladder cell lines and facilitate cell growth. Moreover, TMPO-AS1 could boost migration and invasive features of bladder cancer cells. Expression of TMPO-AS1 has been found to be induced by EBF transcription factor 1 (EBF1). This cytoplasmic lncRNA serves as a sponge for miR-98-5p. EBF1 has been verified to be a target of miR-98-5p whose expression is negatively correlated with expression of miR-98-5p. EBF1 up-regulation restores the suppressive role of TMPO-AS1 silencing in the development of bladder cancer (Luo et al., 2020). Another study in bladder cancer has confirmed the role of TMPO-AS1 in enhancement of cell proliferation, migratory potential, and invasion and suppression of cell. Mechanical studies have also shown that E2F1 up-regulates TMPO-AS1. In addition, TMPO-AS1 has been found to facilitate the interaction between E2F1 and OTUB1. This interaction leads to deubiquitination of E2F1 and its stabilization facilitating the role of TMPO-AS1 in induction of malignant phenotypes in bladder cancer. Further studies have confirmed that TMPO-AS1 induces growth of bladder cancer through an E2F1-dependent manner. This study has verified the importance of a TMPO-AS1/E2F1 positive regulatory circuit in the development of bladder cancer (Zhang et al., 2021).

TMPO-AS1 has also up-regulated in glioma cell lines parallel with down-regulation of miR-383-5p. TMPO-AS1 silencing has intimidated proliferation, migration and invasive abilities of glioma cells. Further experiments have shown that miR-383-5p is a target of TMPO-AS1 (Liu et al., 2020a).

**FIGURE 1:**
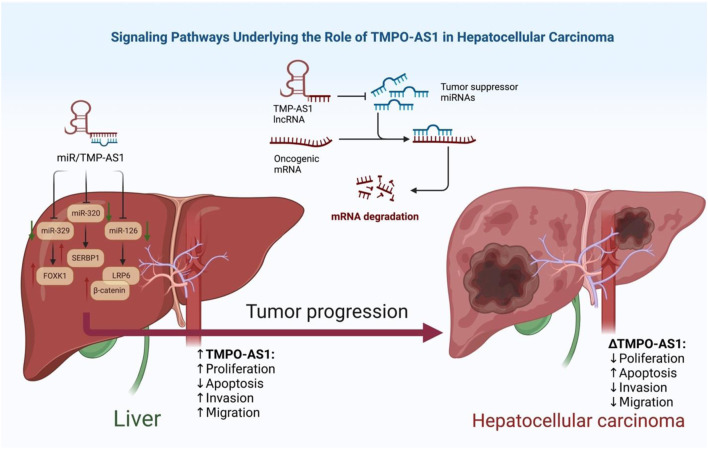
Signaling Pathways underlying the role of TMPO-AS1 in Hepatocellular Carcinoma.

Expression of TMPO-AS1 has also been elevated in hepatocellular carcinoma cell lines. TMPO-AS1 silencing has suppressed viability, migration aptitude and invasiveness of these cells. This lncRNA has mainly located in the cytoplasm of hepatocellular carcinoma cells, where it sponges miR-320a and facilitates up-regulation of SERBP1 (Wang et al., 2020). Another study in this type of cancer has shown that TMPO-AS1 boosts both proliferation and EMT through targeting the miR-126-3p/LRP6/β-catenin axis (Huang et al., 2021) (Figure 1).

In osteosarcoma cells, TMPO-AS1/miR-329/E2F1 axis has been acknowledged as an imporatnt regulator of cell proliferation and apoptosis. Inhibition of TMPO-AS1, overexpression of miR-329 and inhibition of E2F1 could defeat proliferation and invasiveness of osteosarcoma cells and enhance their apoptosis. Moreover, TMPO-AS1 could regulate EMT process in osteosarcoma cells *via* the mentioned axis (Liu et al., 2020b). Another study in this type of cancer has revealed the importance of TMPO-AS1/miR-199a-5p/WNT7B axis in the enhancemnet of tumorigenic properties (Cui and Zhao, 2020).

**FIGURE 2:**
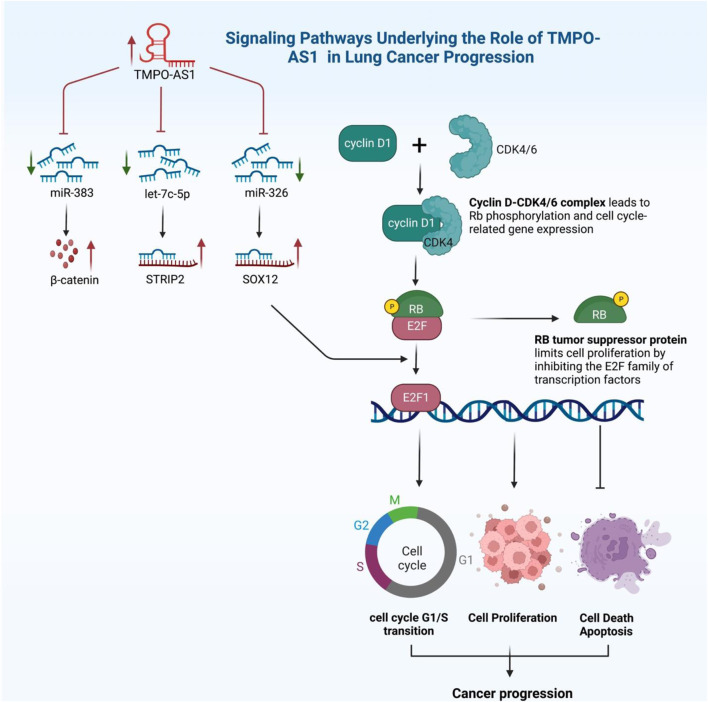
Signaling Pathways underlying the role of TMPO-AS1 in Lung Cancer Progression.

Likewise, Wei and his colleagues showed that E2F1-regulate TMPO-AS1 lncRNA affects lung cancer cell proliferation through controlling the miR-326/SOX12 pathway (Wei et al., 2020). They revealed that advanced clinical stage and poor prognosis in Lung adenocarcinoma (LUAD) were linked to increased TMPO-AS1 expression. Furthermore, reducing TMPO-AS1 expression could slow LUAD cell growth by stopping the cell cycle at the G0/G1 stage and triggering apoptosis. Similarly, Li et al. reported that inhibiting TMPO-AS1 through the miR-143-3p/CDK1 pathway causes increasing apoptosis process in Lung cancer cells (Li et al., 2021) (Figure 2).

Meanwhile, TMPO-AS1/miR-577/RAB14 (Yang et al., 2019) and TMPO-AS1/miR-143-3p/ZEB1 (Gang et al., 2020) axes have been identified as important regulator of malignnat phenotyps of cervical cancer cells.

In breast cancer cell lines, TMPO-AS1 has been found to sponge for miR-4731-5p (Wang et al., 2021), miR-140-5p (Zhu et al., 2021) and miR-1179 (Ning et al., 2021) and up-regulate oncogenic targets of these miRNAs. Besides, TMPO-AS1 has been found to be over-expressed in endocrine therapy-resistant MCF-7 cells compared with estrogen inducible parental cells. Mechanistically, TMPO-AS1 enhances proliferative ability and viability of estrogen receptor (ESR)-positive breast cancer cells. Moreover, expression of this lncRNA is linked with the estrogen signaling cascade. TMPO-AS1 can also up-regulate expression of ESR1 through stabilizing its transcripts. Up-regulation of ESR1 transcript by this lncRNA has a crucial impact in the proliferation of ESR-positive breast cancer (Mitobe et al., 2019).

**FIGURE 3:**
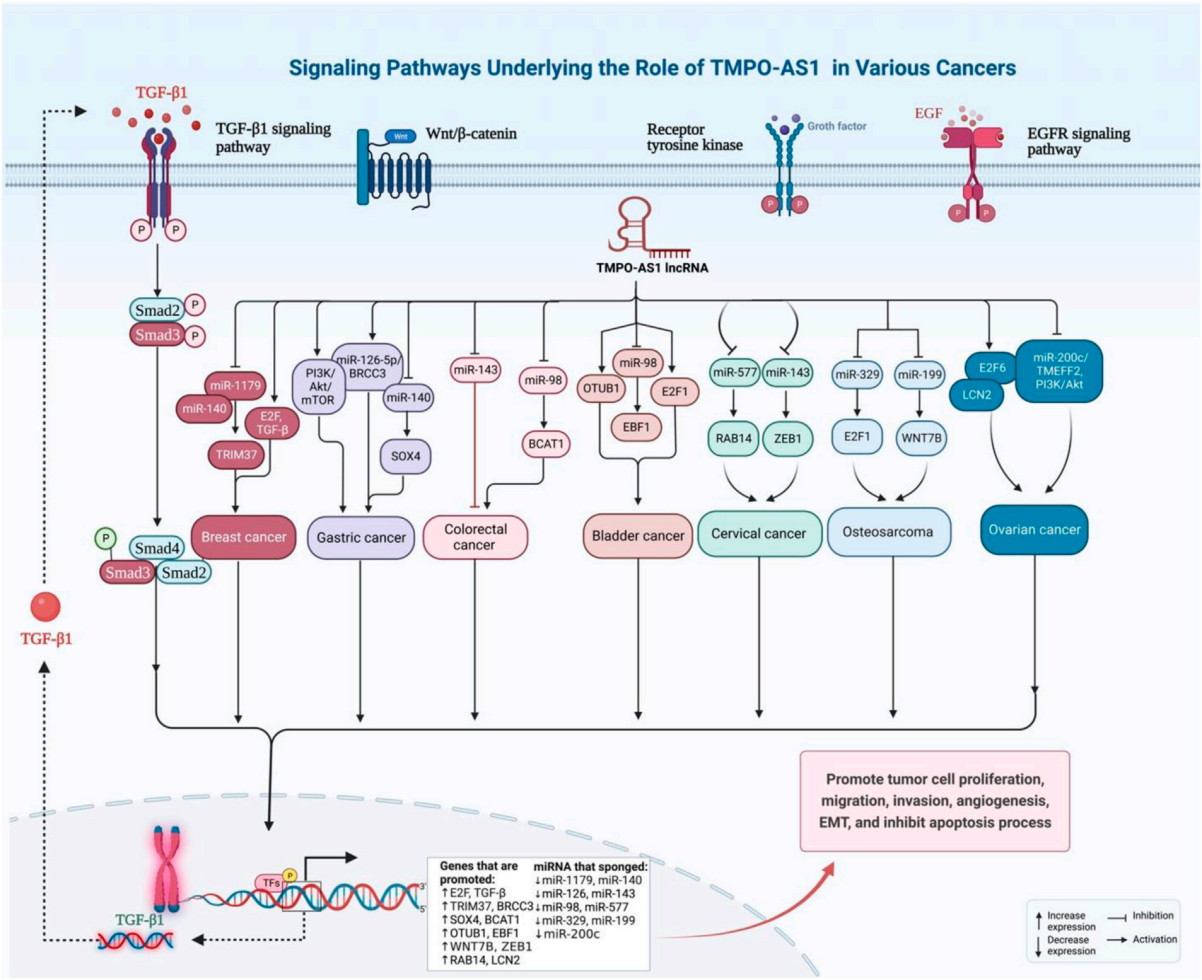
Signaling Pathways underlying the role of TMPO-AS1 in Various Cancers.

In prostate cancer cells, TMPO-AS1 is mainly localized in the cytoplasm and directly down-regulated by androgen receptor. Up-regulation of TMPO-AS1 could increase cell proliferation through enhancing cell cycle progression (Huang et al., 2018) (Table 1). Figure 3 shows the expression pattern, targets and effects of TMPO-AS1 dysregulation in different types of cancer cells.

**TABLE 1:** Expression pattern of TMPO-AS1 in cancer cell lines (Δ: knock-down or deletion, DOC: docetaxel).

Tumor type	Targets/Regulators and signaling pathways	Cell line	Function	References
Nasopharyngeal carcinoma	miR-320a/SOX4	SUNE-1 and C666-1	∆TMPO-AS1 ↓ cell growth ↓ invasion	Xing et al. (2021)
Bladder cancer	EBF1	SV- HUC- 1, T24 UMUC3, 5637, J82	↑ TMPO-AS1 ↑ proliferation ↑ migration ↑ invasion	Luo et al. (2020)
	OTUB1/E2F1	5637, T24, and RT4, BIU87, EJ	∆ TMPO-AS1 ↓ proliferation ↓ migration ↓ invasion ↑ apoptosis	Zhang et al. (2021)
Glioma	miR-383-5p	NHA, U251, A172, LN229	∆ TMPO-AS1 ↓ proliferation ↓ migration ↓ invasion	Liu et al. (2020a)
Hepatocellular carcinoma	miR-320a/SERBP1	HepG2, SNU-387, HCCLM3, SMMC-7721, Huh7, LO2	∆ TMPO-AS1 ↓ proliferation ↓ migration ↓ invasion	Wang et al. (2020)
	miR-329-3p/FOXK1	THLE‐3, Huh7, Hep3B, LM3	∆ TMPO-AS1 ↑ Apoptosis ↓ invasion ↓ migration	Guo and Wang, (2020)
	miR-126-3p/LRP6/β-catenin axis	Hep3B, Huh7, SMMC-7721, Bel-7402, SK-Hep-1, LM9, L-02	∆ TMPO-AS1 ↓ migration ↓ invasion	Huang et al. (2021)
Osteosarcoma	miR-329/E2F1	Saos-2, (HCOs)	↑ TMPO-AS1 ↑ Proliferation ↑ metastasis	Liu et al. (2020b)
	miR-199a‐5p/WNT7B	U20S, MG‐63, SAOS‐2 143B, FOB1.19	∆ TMPO-AS1 ↓ proliferation ↑ apoptosis	Cui and Zhao, (2020)
Cervical cancer	miR-577/RAB14	HeLa, C-33a, SiHa, HCC94	∆ TMPO-AS1 ↓ migration	Yang et al. (2019)
	miR-143-3p/ZEB1	HeLa, SiHa, CaSki, C-33A	↑ TMPO-AS1 ↑ proliferation ↑ migration ↑ invasion	Gang et al. (2020)
Breast cancer	miR-4731-5p	Hs-578T, MCF7, ZR-75–30, HCC1937	∆TMPO-AS1 ↑ apoptosis ↑ cell cycle arrest ↓ migration ↓ invasion	Wang et al. (2021)
	miR-140-5p	MCF7, T47D, MDA-MB-231. SKBR3	∆ TMPO-AS1 ↓ viability ↓ invasion	Zhu et al. (2021)
	miR-1179/TRIM37	MDA-MB-231, MCF7	∆ TMPO-AS1 ↑ sensitivity to DOC ↓ migration	Ning et al. (2021)
Triple negative breast cancer	E2F/TGF‐β	MDA‐MB‐231 and MDA-MB‐468	∆ TMPO-AS1 ↓ migration	Mitobe et al. (2020)
Lung Carcinoma	miR-143-3p	H1299, A549, 95D, H125	↓ TMPO-AS1 ↓ viability ↑ apoptosis	Li et al. (2021)
Lung adenocarcinoma	miR-326 /SOX12/E2F1	HCC827, A549, H838, H1299, SK-LU-1, H23	∆ TMPO-AS1 ↓ proliferation ↑ cell cycle arrest	Wei et al. (2020)
	miR-383-5p	A549, H1299, H1975, H226, PC9, SPC-A,16HBE	↑ TMPO-AS1 ↑ proliferation ↑ invasion	Mu et al. (2020)
	let-7c-5p/STRIP2	h1650, A549, SPC-A1, and H1975, BEAS-2B	↑ TMPO-AS1/let-7c-5p/STRIP2 Adverse outcomes	Wang et al. (2022)
Retinoblastoma	miR-199a-5p/HIF-1α	HXO-RB44 SO-Rb50	↑TMPO-AS1 ↑proliferation	Peng et al. (2020)
Ovarian cancer	LCN2/E2F6	SKOV3, A2780, HO-8910, OVCAR-3, CAOV3	∆ TMPO-AS1 ↓ proliferation ↓ migration ↓ invasion	Zhao et al. (2020a)
	miR-200c /TMEFF2/PI3K/Akt	HOSEpiC, SKOV3	∆ TMPO-AS1 ↓ invasion ↓ drug resistance to 5-FU	Li et al. (2020a)
Non-small cell lung cancer	miR-204-3p/ERBB2	BEAS-2B, A549, H226, H522 and H1299	↑ TMPO-AS1 ↑ Proliferation ↑ migration ↑ invasion	Yu et al. (2020)
	TMPO	95D, A549, H1299, H460, H1975, BESA-2B	∆ TMPO-AS1 ↓ growth ↓ invasion	Qin et al. (2019)
Gastric cancer	miR-126-5p /PI3K/Akt/mTOR pathway/BRCC3	MKN-45, AGS, SGC-7901 SNU-16, GES1	↑ TMPO-AS1 ↑ proliferation ↑ migration ↑ angiogenesis	Hu et al. (2021)
	miR-140-5p/SOX4	GC-27, SGC-7901, BGC-823 AGS, GES1	↑ TMPO-AS1 ↑ proliferation ↑ migration ↑ invasion	Sun and Han, (2020)
Cholangiocarcinoma	let-7g-5p/HMGA1	HCCC9810, HuCCT1, RBE HIBEC	∆ TMPO-AS1 ↓ proliferation ↑ apoptosis	Chang and Yao, (2022)
Pancreatic carcinoma	miR-383-5p/SOX11	HPDE6-C7, SW 1990 PANC-1	∆ TMPO-AS1 ↓ migration ↓ invasion	Xue et al. (2021)
Thyroid cancer	miR-498	TPC-1, IHH-4, A-PTC, CUTC5, nthy-ori3-1	∆ TMPO-AS1 ↑ apoptosis ↓ migration ↓ invasion	Li et al. (2020b)
Colorectal cancer	miR-143-3p	SW480, HCT15, SW1116, HCT116, NCM460	∆ TMPO-AS1 ↓ proliferation ↓ migration ↓ invasion	Zhao et al. (2020b)
	miR-98-5p/BCAT1	HCT15, HT-29, HCT116, SW116, FHC	∆ TMPO-AS1 ↓ proliferation ↑ apoptosis	Ye et al. (2022)
Gallbladder carcinoma	miR-1179/E2F2 axis	GC-996, GBC-SD, EH-GB1, NOZ, H69	∆ TMPO-AS1 ↓ proliferation ↓ migration ↓ invasion ↓ EMT	Sui and Sui, (2021)
Prostate cancer	AR	LNCaP, DU145, 22Rv1, PC-3 WPMY	↑TMPO-AS1 ↑proliferation ↓apoptosis	Huang et al. (2018)
Esophageal cancer	miR‐498	EC109 and KYSE70	↑ TMPO-AS1 ↓ propofol effect on EMT	Gao et al. (2020)
Esophageal squamous cell carcinoma	FUS, p300	Het-1A, NE-1 HEK293T,KYSE30, KYSE150,KYSE180, KYSE410,KYSE510, KYSE520	∆TMPO-AS1 ↓ proliferation ↓ migration ↓ invasion	Luo et al. (2022)
Endometrial cancer	miR-140 & miR-143/GLUT1	Ishikawa and HHUA	∆TMPO-AS1 ↓ Glycolysis ↓ resistance to Paclitaxel therapy	Dong et al. (2022)

### Animal studies

Results of studies in xenograft models of different cancer consistently support the oncogenic role of TMPO-AS1 (Table 2). All studies have confirmed that TMPO-AS1 silencing results in reduction of tumor burden in animal models. A single study in xenograft model of nasopharyngeal carcinoma SUNE1 cells has also verified the inhibitory effect of TMPO-AS1 knockdown on nasopharyngeal carcinoma cells growth. A notable reduction has been observed in tumor volume in the mice group injected with sh-TMPO-AS1. Based on the immunohistochemistry staining and qRT-PCR assays, substantial suppression in the expression of SOX4 and significant increase in miR-320a expression have been observed in mice injected with sh-TMPO-AS1-transfected cells. *In vivo* rescue experiments have also confirmed the sponging effect of TMPO-AS1 on miR-320a. miR-320a mimics-transfected SNUE1 cells have exhibited lower *in vivo* growth. Besides, TMPO-AS1 over-expression has reduced miR-320a levels in tumor tissues elevated by miR-320a mimics transfection (Xing et al., 2021).

**TABLE 2:** Influence of TMPO-AS1 in carcinogenesis based on studies in animal models (NOD-SCID: non-obese diabetic/severe combined immunodeficiency, Δ: knock-down or deletion).

Tumor type	Animal models	Results	Related pathways/targets	References
Nasopharyngeal carcinoma	BALB/c nude mice	∆TMPO-AS1 ↓Tumor growth	miR-320a/SOX4 axis	Xing et al. (2021)
Hepatocellular carcinoma	BALB/c nude mice	∆TMPO-AS1 ↓tumor growth	miR-320a/SERBP1 axis	Wang et al. (2020)
	male BALB/c athymic nude mice	∆TMPO-AS1 ↓tumor growth ↓tumor size	miR-329-3p/FOXK1 axis	Guo and Wang, (2020)
Breast cancer	BALB/c nude mice	∆TMPO-AS1 ↓tumor growth	miR-140-5p	Zhu et al. (2021)
	Female nude mice	∆TMPO-AS1 ↓hormone-refractory tumor growth	ESR1	Mitobe et al. (2019)
Ovarian cancer	female BALB/c nude mice	∆TMPO-AS1 ↓Tumorigenesis ↓angiogenesis	LCN2/E2F6	Zhao et al. (2020a)
	female BALB/C nude mice	∆TMPO-AS1 ↓tumor growth	miR-200c /TMEFF2/PI3K/Akt axis	Li et al. (2020a)
Pancreatic carcinoma	BALB/c/nude mice	↓TMPO-AS1 ↓tumor growth	miR-383-5p	Xue et al. (2021)
Thyroid cancer	Nude male mice	↓TMPO-AS1 ↓tumor growth	miR-498	Li et al. (2020b)
Bladder cancer	male BALB/c nude mice	∆TMPO-AS1 ↓tumor growth	OTUB1/E2F1	Zhang et al. (2021)
Lung adenocarcinoma	Nude mice	∆TMPO-AS1 ↓tumor growth	miR-383-5p	Mu et al. (2020)
Colorectal cancer	Female BALB/c nude mice	∆TMPO-AS1 ↓tumor growth	miR-143-3p	Zhao et al. (2020b)
Triple-negative breast cancer	NOD/SCID mice	∆TMPO-AS1 ↓metastasis ↓tumor growth	E2F/TGFβ	Mitobe et al. (2020)
Non-small cell lung cancer	male BALB/C nude mice	∆TMPO-AS1 ↓tumor weight	TMPO	Qin et al. (2019)
Cholangiocarcinoma	female BALB/c nude mice	∆TMPO-AS1 ↓tumor growth	let-7g-5p/high-mobility group A1	Chang and Yao, (2022)
Esophageal squamous cell carcinoma	female BALB/c nu/nu mice	∆TMPO-AS1 ↓lymph node metastasis	FUS/p300	Luo et al. (2022)

Inoculation of stably transfected RT4 bladder cancer cells into nude mice has also verified that TMPO-AS1 can enhance bladder cancer growth through E2F1 *in vivo*. TMPO-AS1 silencing has substantially suppressed tumor growth, while up-regulation of E2F1 has eliminated the inhibitory effects on tumor growth resulted from TMPO-AS1 silencing. Besides, the immunohistochemistry staining has shown that TMPO-AS1 silencing results in a considerable reduction in Ki-67 and E2F1 levels and a prominent increase in caspase-3 expression (Zhang et al., 2021). Additionally, TMPO-AS1 has been shown to enhance proliferative ability and viability of ESR-positive breast cancer cells in animal models (Mitobe et al., 2019). Moreover, its silencing can decrease hormone-refractory tumor growth (Mitobe et al., 2019). In addition, experiments in animal models of ovarian cancer have confirmed a significant decrease in the angiogenic potential following TMPO-AS1 silencing (Zhao et al., 2020a). Finally, in animal models of esophageal squamous cell carcinoma, TMPO-AS1 silencing can suppress lymph node metastasis (Luo et al., 2022).

## Studies in clinical samples

Expression of TMPO-AS1 has been found to be elevated in all kinds of examined cancerous clinical samples (Table 3). In hepatocellular carcinoma samples, over-expression of TMPO-AS1 has been related to advanced stages and worse prognosis (Wang et al., 2020). Over-expression of TMPO‐AS1 has also been related to large tumor size, lymphatic metastasis, and advanced stage in another study on patients with hepatocellular carcinoma (Guo and Wang, 2020). Meanwhile, portal vein tumor thrombosis has been another feature that has been associated with up-regulation of this lncRNA in hepatocellular carcinoma (Huang et al., 2021). *In silico* analysis of TCGA database and expression assays in clinical specimens of bladder cancer have confirmed up-regulation of TMPO-AS1 in bladder cancer tissues compared with normal bladder samples. Notably, worse survival has been reported for patients with over-expression of this lncRNA. Moreover, up-regulation of TMPO-AS1 has been correlated with muscle invasiveness and advance TNM stage in these patients (He et al., 2020). RNA-sequencing data of breast cancer samples has shown correlation between TMPO-AS1 level and proliferative biomarkers. Moreover, TMPO-AS1 positivity has been significantly correlated with poor prognosis of patients with this type of cancer (Mitobe et al., 2019). TMPO-AS1 has also been suggested to be a valuable diagnostic and prognostic marker for prostate cancer, since its up-regulation has been associated with poorer prognosis of patients with prostate cancer. *In silico* studies have predicated associations between TMPO-AS1 and a number of biological processes participating in the progression of prostate cancer (Huang et al., 2018). Similar to other types of cancer, up-regulation of TMPO-AS1 has been associated with lymph node involvement and distant metastasis in patients with colorectal cancer (Mohammadrezakhani et al., 2020).

**TABLE 3:** Dysregulation of TMPO-AS1 or other genes that interact with TMPO-AS1 in clinical specimens (PANTs: paired adjacent normal tissues, OS: overall survival, LNM: lymph node metastasis, BCR: biochemical recurrence).

Tumor type	Samples	Expression of TMPO-AS1 or other genes (tumor vs Normal)	Cancer/TNM stage	Kaplan-meier analysis (impact of TMPO-AS1 dysregulation)	Univariate/Multivariate cox regression	Association of TMPO-AS1 expression with clinicopathologic features	Related pathways/targets	References
Nasopharyngeal carcinoma (NPC)	45 NPC tissue samples and 22 normal nasopharynx tissues	Upregulated (which sponges miR320-a)	I-IV	-	-	Associated with metastasis and advanced clinical stage	miR-320a/SOX4 axis	Xing et al. (2021)
Bladder cancer (BC)	40 fresh cancer tissues and PANTs	Upregulated (which sponges miR-98-5p)	T1-T4	Poor OS	Independent prognostic factor	-	EBF1	Luo et al. (2020)
	6 cancerous and PANTs	Upregulated	I-IV	Poor OS	-	Associated with recurrence of BC + advanced tumor stage	OTUB1/E2F1	Zhang et al. (2021)
Hepatocellular carcinoma (HCC)	42 HCC samples and PANTs	Upregulated	I-IV	Poor OS	-	Associated with TNM stage and metastasis	miR-320a/SERBP1 axis	Wang et al. (2020)
	48 HCC samples and PANTs	Upregulated	I-IV	Poor OS	-	Associated with Tumor size, Lymphatic metastasis, TNM	miR-329-3p/FOXK1 axis	Guo and Wang, (2020)
	53 HCC samples and PANTs	Upregulated	-	Poor OS	-	Associated with TNM stages, portal vein tumor thrombosis	miR-126-3p/LRP6/β-catenin axis	Huang et al. (2021)
Osteosarcoma	51 samples of cancer tissues and PANTs	Upregulated	-	-	-	-	miR-329/E2F1 axis	Liu et al. (2020b)
	56 cancer tissues and PANTs	Upregulated (which sponges miR-199a‐5p)	-	-	-	-	miR-199a‐5p/WNT7B axis	Cui and Zhao, (2020)
Breast cancer	22 cancer tissues and PANTs	Upregulated (which sponges miR-4731-5p)	I-IV	Poor OS	-	-	miR-4731-5p	Wang et al. (2021)
	40 Breast cancer tissues+ 15 healthy controls	Upregulated (which sponges miR-140-5p)	-	-	-	-	miR-140-5p	Zhu et al. (2021)
	115 breast cancer tissues	Upregulation resulted in poor prognosis	I-III	Poor OS	Prognostic factor for OS and distant disease-free survival	Associated with stage, pathological T factor, histological grade and HER2 status	ESR1	Mitobe et al. (2019)
Laryngeal squamous cell carcinoma (LSCC)	187 cancer tissues and PANTs	Upregulated	I-IV	Poor OS	Independent prognostic biomarker for LSCC patients	Associated with clinical stage, LNM	-	(Zhang et al., 19922020)
Lung Carcinoma (LC)	50 cancer tissues and PANTs	Upregulated	-	Poor OS	-	-	miR-143-3p	Li et al. (2021)
Lung adenocarcinoma (LUAD)	25 cancer tissues and PANTs + GEPIA	Upregulated	I-IV	Poor OS	-	Associated with TNM stage, LMN and high risk of mortality	miR-326/SOX12/E2F1 axis	Wei et al. (2020)
	6 cancer tissues and PANTs	Upregulated	-	Poor OS	-	-	miR-383-5p	Mu et al. (2020)
Retinoblastoma	tissue samples from 33 children + normal retinal pigment epithelial tissue	Upregulated	A-E (Based on tumor progression)	-	-	-	HIF-1α/miR-199a-5p	Peng et al. (2020)
Cholangiocarcinoma	36 cancer tissues and PANTs	Upregulated	I-IV	-	-	-	let-7g-5p/HMGA1	Chang and Yao, (2022)
Ovarian cancer	86 cancer tissues and PANTs	Upregulated (promotes LCN2)	I-IV/G1-G3 (Fuhrman)	Poor OS	-	Associated with TNM stage, Fuhrman grade and tumor size	LCN2/E2F6	Zhao et al. (2020a)
Non-small cell lung cancer	30 cancer tissues and PANTs	Upregulated (which Sponges miR-204-3p)	-	Poor OS	-	-	miR-204-3p/ERBB2 axis	Yu et al. (2020)
	40 cancer tissues and PANTs	Upregulated	I-III	Poor OS	Lymph node metastasis is an independent prognostic factor	Associated with TNM stage and LMN	TMPO	Qin et al. (2019)
Gastric cancer	70 cancer tissues and PANTs	Upregulated (which Sponges miR-126-5p)	I-IV	Poor OS	-	Associated with TNM stage and LMN	miR-126-5p/PI3K/Akt/mTOR pathway/BRCC3	Hu et al. (2021)
	105 cancer tissues and PANTs	Upregulated (which Sponges miR-140-5p)	I-IV	Poor OS	-	Associated with larger tumor size and advanced TNM stage	miR-140-5p/SOX4 axis	Sun and Han, (2020)
Pancreatic carcinoma	38 cancer tissues and PANTs	Upregulated	-	-	-	-	miR-383-5p/SOX11 axis	Xue et al. (2021)
Thyroid cancer	40 cancer tissues and PANTs	Upregulated	-	-	-	-	miR-498	Li et al. (2020b)
Gallbladder carcinoma (GBC)	30 cancer tissues and PANTs	Upregulated	I-IV	Poor OS	Poor OS	-	miR-1179 /E2F2 axis	Sui and Sui, (2021)
Colorectal cancer	50 cancer tissues and PANTs	Upregulated	I-IV	-	-	Associated with LMN and distant metastasis	-	Mohammadrezakhani et al. (2020)
Prostate cancer	54 cancer tissues and PANTs	Upregulated	pT2a-pT4	Higher BCR	-	Associated with Gleason score, pathological stage, pathological stage and PSA level	AR	Huang et al. (2018)
Esophageal squamous cell carcinoma	108 samples + TCGA dataset	Upregulated	I-IV	Poor OS	-	-	FUS/p300	Luo et al. (2022)

Diagnostic role of TMPO-AS1 has been assessed in osteosarcoma and colorectal cancer, yielding better performance in the former type of cancer (Table 4).

**TABLE 4:** Diagnostic value of TMPO-AS1 in cancers.

Tumor type	Samples	Distinguish between	Area under curve	Sensitivity (%)	Specificity (%)	References
Osteosarcoma	51 pairs of cancer tissues and adjacent tissues	Osteosarcoma tissues vs control tissues	0.8	70.59	82.35	Liu et al. (2020b)
Colorectal cancer	21 tumor samples and 21 margin samples	Tumor vs margin samples	0.6973			Mohammadrezakhani et al. (2020)

## Concluding remarks

TMPO-AS1 is an lncRNA with crucial roles in the carcinogenic processes. The best appreciated route of participation of TMPO-AS1 in these processes is its function as a molecular sponge for miRNAs. This lncRNA serves as a sponge for miR-383-5p, miR-320a, miR-329-3p, miR-126, miR-329, miR‐199a‐5p, miR-577, miR-4731-5p, miR-140-5p, miR-1179, miR-143-3p, miR-326, miR-383-5p, let-7c-5p, let-7g-5p, miR-199a-5p, miR-200c, miR-204-3p, miR-126-5p, miR-383-5p, miR-498, miR-143-3p, miR-98-5p, miR-140 and miR-143. Most of these miRNAs have anti-cancer effects through modulation of cell apoptosis, survival and differentiation. Thus, TMPO-AS1 has several routes of actions. Each of TMPO-AS1/miRNA axes has the potential to be used as diagnostic marker or therapeutic target. However, those being dysregulated in more than on type of cancer seem to be more appropriate, since they can be used in different types of cancers. Moreover, TMPO-AS1/miRNA/mRNA axes having specific roles in a certain type of cancer can be used for diagnostic marker for this type of cancer, particularly in the follow-up of patients after conduction of therapeutic modalities.

Several studies have reported regulatory role of TMPO-AS1 on PI3K/Akt/mTOR pathway. Based on the importance of this pathway in cancer progression and availability of targeted therapies against this pathway (Alzahrani, 2019), therapeutic modalities that affect expression of TMPO-AS1 are promising strategies for enhancement of the effects of PI3K/Akt/mTOR-targeting modalities.

Although dysregulation of TMPO-AS1 has been described in several cancers, diagnostic role of this lncRNA has only been assessed in two types of cancerous tissues *versus* non-cancerous tissues. Moreover, its application as a diagnostic marker in the peripheral blood has not been evaluated. Since assessment of expression profile of lncRNAs in the peripheral blood can facilitate identification of novel strategies for non-invasive detection of malignant conditions, further studies should evaluate expression of TMPO-AS1 in different stages of cancer progression to find its potential as early diagnostic marker and its relevance with progression of cancer. Based on the heterogeneity of expression profiles in the cancerous samples, a more applicable strategy is identification of panels of lncRNAs which can discriminate cancer patients from healthy controls with higher efficacy.

Several experiments have shown that TMPO-AS1 silencing can attenuate malignant behavior of cancer cells in cultures and in xenograft models of cancer. Thus, TMPO-AS1-targeting strategies have the potential to be used as therapeutic modalities for cancer treatment. Therefore, future investigations should find effective methods for specific delivery of anti-TMPO-AS1 modalities to cancer cells and evaluate their safety and efficacy in suppression of tumor growth in clinical settings.

Finally, preliminary studies have shown that TMPO-AS1 silencing can enhance sensitivity to paclitaxel (Dong et al., 2022) and docetaxel (Ning et al., 2021) in endometrial and breast cancers, respectively. Thus, targeted therapies against this lncRNA are promising strategies in defeating resistance to chemotherapy. Future studies are necessary to compare expression levels of this lncRNA between patients who are response to certain chemotherapeutic agents and unresponsive ones to elaborate the effect of this lncRNA in chemoresistance.

## References

[B1] Alzahrani A. S. (2019). PI3K/Akt/mTOR inhibitors in cancer: At the bench and bedside. Semin. Cancer Biol. 59, 125–132. 10.1016/j.semcancer.2019.07.009 31323288

[B2] Chang H. Yao Y. (2022). lncRNA TMPO antisense RNA 1 promotes the malignancy of cholangiocarcinoma cells by regulating let-7g-5p/high-mobility group A1 axis. Bioengineered 13 (2), 2889–2901. 10.1080/21655979.2022.2025700 35040749 PMC8973948

[B3] Cui H. Zhao J. (2020). LncRNA TMPO-AS1 serves as a ceRNA to promote osteosarcoma tumorigenesis by regulating miR-199a-5p/WNT7B axis. J. Cell. Biochem. 121 (3), 2284–2293. 10.1002/jcb.29451 31680323

[B4] Dong P. Wang F. Taheri M. Xiong Y. Ihira K. Kobayashi N. (2022). Long non-coding RNA TMPO-AS1 promotes GLUT1-mediated glycolysis and paclitaxel resistance in endometrial cancer cells by interacting with miR-140 and miR-143. Front. Oncol. 12, 912935. 10.3389/fonc.2022.912935 35712514 PMC9195630

[B5] Frankish A. Diekhans M. Ferreira A. M. Johnson R. Jungreis I. Loveland J. (2019). GENCODE reference annotation for the human and mouse genomes. Nucleic Acids Res. 47 (D1), D766-D773. 10.1093/nar/gky955 30357393 PMC6323946

[B6] Gang X. Yuan M. Zhang J. (2020). Long non-coding RNA TMPO-AS1 promotes cervical cancer cell proliferation, migration, and invasion by regulating miR-143-3p/ZEB1 Axis. Cancer Manag. Res. 12, 1587–1599. 10.2147/CMAR.S226409 32184662 PMC7060785

[B7] Gao M. Guo R. Lu X. Xu G. Luo S. (2020). Propofol suppresses hypoxia-induced esophageal cancer cell migration, invasion, and EMT through regulating lncRNA TMPO-AS1/miR-498 axis. Thorac. Cancer 11 (9), 2398–2405. 10.1111/1759-7714.13534 32643321 PMC7471028

[B8] Ghaforui‐Fard S. Vafaee R. Taheri M. (2019). Taurine‐upregulated gene 1: A functional long noncoding RNA in tumorigenesis. J. Cell. Physiol. 234 (10), 17100–17112. 10.1002/jcp.28464 30912122

[B9] Ghafouri-Fard S. Azimi T. Hussen B. M. Abak A. Taheri M. Dilmaghani N. A. (2021). Non-coding RNA activated by DNA damage: Review of its roles in the carcinogenesis. Front. Cell Dev. Biol. 9, 714787. 10.3389/fcell.2021.714787 34485302 PMC8415109

[B10] Ghafouri-Fard S. Dashti S. Taheri M. (2020). The HOTTIP (HOXA transcript at the distal tip) lncRNA: Review of oncogenic roles in human. Biomed. Pharmacother. 127, 110158. 10.1016/j.biopha.2020.110158 32335298

[B11] Ghafouri-Fard S. Taheri M. (2019). Maternally expressed gene 3 (MEG3): A tumor suppressor long non coding RNA. Biomed. Pharmacother. 118, 109129. 10.1016/j.biopha.2019.109129 31326791

[B12] Guo X. Wang Y. (2020). LncRNA TMPO-AS1 promotes hepatocellular carcinoma cell proliferation, migration and invasion through sponging miR-329-3p to stimulate FOXK1-mediated AKT/mTOR signaling pathway. Cancer Med. 9 (14), 5235–5246. 10.1002/cam4.3046 32462698 PMC7367632

[B13] He Y. C. Bi Y. G. Jiang L. (2020). LncRNA TMPO-AS1 promotes proliferation and migration in bladder cancer. Eur. Rev. Med. Pharmacol. Sci. 24 (17), 8740–8746. 10.26355/eurrev_202009_22812 32964962

[B14] Hon C. C. Ramilowski J. A. Harshbarger J. Bertin N. Rackham O. J. Gough J. (2017). An atlas of human long non-coding RNAs with accurate 5' ends. Nature 543 (7644), 199–204. 10.1038/nature21374 28241135 PMC6857182

[B15] Hu Y. Zhang Y. Ding M. Xu R. (2021). Long noncoding RNA TMPO-AS1/miR-126-5p/BRCC3 axis accelerates gastric cancer progression and angiogenesis via activating PI3K/Akt/mTOR pathway. J. Gastroenterol. Hepatol. 36 (7), 1877–1888. 10.1111/jgh.15362 33295056

[B16] Huang W. Chen Q. Dai J. Zhang Y. Yi Y. Wei X. (2021). Long noncoding TMPO antisense RNA 1 promotes hepatocellular carcinoma proliferation and epithelial-mesenchymal transition by targeting the microRNA-126-3p/LRP6/β-catenin axis. Ann. Transl. Med. 9 (22), 1679. 10.21037/atm-21-5593 34988188 PMC8667144

[B17] Huang W. Su X. Yan W. Kong Z. Wang D. Huang Y. (2018). Overexpression of AR-regulated lncRNA TMPO-AS1 correlates with tumor progression and poor prognosis in prostate cancer. Prostate 78 (16), 1248–1261. 10.1002/pros.23700 30105831

[B18] Kung J. T. Colognori D. Lee J. T. (2013). Long noncoding RNAs: Past, present, and future. Genetics 193 (3), 651–669. 10.1534/genetics.112.146704 23463798 PMC3583990

[B19] Li H. Zhou Y. Cheng H. Tian J. Yang S. (2020). Roles of a TMPO-AS1/microRNA-200c/TMEFF2 ceRNA network in the malignant behaviors and 5-FU resistance of ovarian cancer cells. Exp. Mol. Pathol. 115, 104481. 10.1016/j.yexmp.2020.104481 32497621

[B20] Li Q. Bian Y. Li Q. (2021). Down-regulation of TMPO-AS1 induces apoptosis in lung carcinoma cells by regulating miR-143-3p/CDK1 Axis. Technol. Cancer Res. Treat. 20, 1533033820948880. 10.1177/1533033820948880 33685293 PMC8093611

[B21] Li Z. Feng Y. Zhang Z. Cao X. Lu X. (2020). TMPO-AS1 promotes cell proliferation of thyroid cancer via sponging miR-498 to modulate TMPO. Cancer Cell Int. 20, 294. 10.1186/s12935-020-01334-4 32669970 PMC7346673

[B22] Liu G. Yang H. Cao L. Han K. Li G. (2020). LncRNA TMPO-AS1 promotes proliferation and invasion by sponging miR-383-5p in glioma cells. Cancer Manag. Res. 12, 12001–12009. 10.2147/CMAR.S282539 33262650 PMC7696628

[B23] Liu X. Wang H. Tao G. L. Chu T. B. Wang Y. X. Liu L. (2020). LncRNA-TMPO-AS1 promotes apoptosis of osteosarcoma cells by targeting miR-329 and regulating E2F1. Eur. Rev. Med. Pharmacol. Sci. 24 (21), 11006–11015. 10.26355/eurrev_202011_23585 33215415

[B24] Luo H. Yang L. Liu C. Wang X. Dong Q. Liu L. (2020). TMPO-AS1/miR-98-5p/EBF1 feedback loop contributes to the progression of bladder cancer. Int. J. Biochem. Cell Biol. 122, 105702. 10.1016/j.biocel.2020.105702 32087328

[B25] Luo X. J. He M. M. Liu J. Zheng J. B. Wu Q. N. Chen Y. X. (2022). LncRNA TMPO-AS1 promotes esophageal squamous cell carcinoma progression by forming biomolecular condensates with FUS and p300 to regulate TMPO transcription. Exp. Mol. Med. 54 (6), 834–847. 10.1038/s12276-022-00791-3 35760875 PMC9243820

[B26] Mitobe Y. Ikeda K. Sato W. Kodama Y. Naito M. Gotoh N. (2020). Proliferation-associated long noncoding RNA, TMPO-AS1, is a potential therapeutic target for triple-negative breast cancer. Cancer Sci. 111 (7), 2440–2450. 10.1111/cas.14498 32437068 PMC7385350

[B27] Mitobe Y. Ikeda K. Suzuki T. Takagi K. Kawabata H. Horie-Inoue K. (2019). ESR1-Stabilizing long noncoding RNA TMPO-AS1 promotes hormone-refractory breast cancer progression. Mol. Cell. Biol. 39 (23), e002611-e319. 10.1128/MCB.00261-19 PMC6851347 31501276

[B28] Mohammadrezakhani H. Baradaran B. Shanehbandi D. Asadi M. Hashemzadeh S. Hajiasgharzadeh K. (2020). Overexpression and clinicopathological correlation of long noncoding RNA TMPO-AS1 in colorectal cancer patients. J. Gastrointest. Cancer 51 (3), 952–956. 10.1007/s12029-019-00333-7 31768869

[B29] Mu X. Wu H. Liu J. Hu X. Wu H. Chen L. (2020). Long noncoding RNA TMPO-AS1 promotes lung adenocarcinoma progression and is negatively regulated by miR-383-5p. Biomed. Pharmacother. 125, 109989. 10.1016/j.biopha.2020.109989 32062549

[B30] Ning X. Zhao J. He F. Yuan Y. Li B. Ruan J. (2021). Long non-coding RNA TMPO-AS1 facilitates chemoresistance and invasion in breast cancer by modulating the miR-1179/TRIM37 axis. Oncol. Lett. 22 (1), 500. 10.3892/ol.2021.12761 33981362 PMC8108256

[B31] Peng X. Yan J. Cheng F. (2020). LncRNA TMPO-AS1 up-regulates the expression of HIF-1α and promotes the malignant phenotypes of retinoblastoma cells via sponging miR-199a-5p. Pathol. Res. Pract. 216 (4), 152853. 10.1016/j.prp.2020.152853 32139259

[B32] Qin Z. Zheng X. Fang Y. (2019). Long noncoding RNA TMPO-AS1 promotes progression of non-small cell lung cancer through regulating its natural antisense transcript TMPO. Biochem. Biophys. Res. Commun. 516 (2), 486–493. 10.1016/j.bbrc.2019.06.088 31230752

[B33] Schmitt A. M. Chang H. Y. (2017). Long noncoding RNAs: At the intersection of cancer and chromatin Biology. Cold Spring Harb. Perspect. Med. 7 (7), a026492. 10.1101/cshperspect.a026492 28193769 PMC5495049

[B34] Sui Z. Sui X. (2021). Long non-coding RNA TMPO-AS1 promotes cell proliferation, migration, invasion and epithelial-to-mesenchymal transition in gallbladder carcinoma by regulating the microRNA-1179/E2F2 axis. Oncol. Lett. 22 (6), 855. 10.3892/ol.2021.13116 34777589 PMC8581476

[B35] Sun Y. Han C. (2020). Long non-coding RNA TMPO-AS1 promotes cell migration and invasion by sponging miR-140-5p and inducing SOX4-mediated EMT in gastric cancer. Cancer Manag. Res. 12, 1261–1268. 10.2147/CMAR.S235898 32110100 PMC7039077

[B36] Thapar R. (2018). Regulation of DNA double-strand break repair by non-coding RNAs. Mol. (Basel, Switz. 23 (11), E2789. 10.3390/molecules23112789 PMC6278438 30373256

[B37] Wang J. Yuan Y. Tang L. Zhai H. Zhang D. Duan L. (2022). Long non-coding RNA-TMPO-AS1 as ceRNA binding to let-7c-5p upregulates STRIP2 expression and predicts poor prognosis in lung adenocarcinoma. Front. Oncol. 12, 921200. 10.3389/fonc.2022.921200 35774125 PMC9237420

[B38] Wang Y. Ma J. Li R. Gao X. Wang H. Jiang G. (2021). LncRNA TMPO-AS1 serves as a sponge for miR-4731-5p modulating breast cancer progression through FOXM1. Am. J. Transl. Res. 13 (10), 11094–11106. 34786045 PMC8581887

[B39] Wang Z. Huang D. Huang J. Nie K. Li X. Yang X. (2020). lncRNA TMPO-AS1 exerts oncogenic roles in HCC through regulating miR-320a/SERBP1 Axis. Onco. Targets. Ther. 13, 6539–6551. 10.2147/OTT.S250355 32753892 PMC7342364

[B40] Wei L. Liu Y. Zhang H. Ma Y. Lu Z. Gu Z. (2020). TMPO-AS1, a novel E2F1-regulated lncRNA, contributes to the proliferation of lung adenocarcinoma cells via modulating miR-326/SOX12 Axis. Cancer Manag. Res. 12, 12403–12414. 10.2147/CMAR.S269269 33293866 PMC7719338

[B41] Xue F. Song X. Zhang S. Niu M. Cui Y. Wang Y. (2021). Long non-coding RNA TMPO-AS1 serves as a tumor promoter in pancreatic carcinoma by regulating miR-383-5p/SOX11. Oncol. Lett. 21 (4), 255. 10.3892/ol.2021.12517 33664819 PMC7882873

[B42] Xing B. Qiao X. F. Qiu Y. H. Li X. (2021). TMPO-AS1 regulates the aggressiveness-associated traits of nasopharyngeal carcinoma cells through sponging miR-320a. Cancer Manag. Res. 13, 415–425. 10.2147/CMAR.S285113 33488123 PMC7815083

[B43] Yang J. Liang B. Hou S. (2019). TMPO-AS1 promotes cervical cancer progression by upregulating RAB14 via sponging miR-577. J. Gene Med. 21 (11), e3125. 10.1002/jgm.3125 31483914

[B44] Ye J. Yan Y. Xin L. Liu J. Tang T. Bao X. (2022). Long non-coding RNA TMPO-AS1 facilitates the progression of colorectal cancer cells via sponging miR-98-5p to upregulate BCAT1 expression. J. Gastroenterol. Hepatol. 37 (1), 144–153. 10.1111/jgh.15657 34370878

[B45] Yu X. Lin Q. Liu F. Yang F. Mao J. Chen X. (2020). LncRNA TMPO-AS1 facilitates the proliferation and metastasis of NSCLC cells by up-regulating ERBB2 via sponging miR-204-3p. Int. J. Immunopathol. Pharmacol. 34, 2058738420958947. 10.1177/2058738420958947 32969763 PMC7520928

[B46] Zhang L. Zhang Y. Zhang C. Hou Y. Tian F. (2020). TMPO-AS1 is an independent prognostic factor for patients with laryngeal squamous cell carcinoma. Rev. Assoc. Med. Bras. 66 (6), 784–788. 10.1590/1806-9282.66.6.784 32696866

[B47] Zhang Y. Zhu Y. Xiao M. Cheng Y. He D. Liu J. (2021). The long non-coding RNA TMPO-AS1 promotes bladder cancer growth and progression via OTUB1-induced E2F1 deubiquitination. Front. Oncol. 11, 643163. 10.3389/fonc.2021.643163 33816295 PMC8013732

[B48] Zhao H. Ding F. Zheng G. (2020). LncRNA TMPO-AS1 promotes LCN2 transcriptional activity and exerts oncogenic functions in ovarian cancer. Faseb J. 34 (9), 11382–11394. 10.1096/fj.201902683R 32692467

[B49] Zhao L. Li Y. Song A. (2020). Inhibition of lncRNA TMPO-AS1 suppresses proliferation, migration and invasion of colorectal cancer cells by targeting miR-143-3p. Mol. Med. Rep. 22 (4), 3245–3254. 10.3892/mmr.2020.11427 32945436 PMC7453500

[B50] Zhu D. Lv W. Zhou X. He Y. Yao H. Yu Y. Zhang G. Zhang Q., (2021). Long non-coding RNA TMPO-AS1 promotes tumor progression via sponging miR-140-5p in breast cancer. Exp. Ther. Med. 21 (1), 17. 10.3892/etm.2020.9449 33235626 PMC7678596

